# Directed physiological networks in the human prefrontal cortex at rest and post transcranial photobiomodulation

**DOI:** 10.1038/s41598-024-59879-7

**Published:** 2024-05-03

**Authors:** Sadra Shahdadian, Xinlong Wang, Hanli Liu

**Affiliations:** Department of Bioengineering, University of Texas at Arlington, Arlington, TX 76019 USA

**Keywords:** Generalized partial directed coherence, Infra-slow oscillation, Neurovascular coupling, Neurometabolic coupling, Transcranial photobiomodulation, Neuroscience, Physiology, Optics and photonics

## Abstract

Cerebral infra-slow oscillation (ISO) is a source of vasomotion in endogenic (E; 0.005–0.02 Hz), neurogenic (N; 0.02–0.04 Hz), and myogenic (M; 0.04–0.2 Hz) frequency bands. In this study, we quantified changes in prefrontal concentrations of oxygenated hemoglobin (Δ[HbO]) and redox-state cytochrome *c* oxidase (Δ[CCO]) as hemodynamic and metabolic activity metrics, and electroencephalogram (EEG) powers as electrophysiological activity, using concurrent measurements of 2-channel broadband near-infrared spectroscopy and EEG on the forehead of 22 healthy participants at rest. After preprocessing, the multi-modality signals were analyzed using generalized partial directed coherence to construct unilateral neurophysiological networks among the three neurophysiological metrics (with simplified symbols of HbO, CCO, and EEG) in each E/N/M frequency band. The links in these networks represent neurovascular, neurometabolic, and metabolicvascular coupling (NVC, NMC, and MVC). The results illustrate that the demand for oxygen by neuronal activity and metabolism (EEG and CCO) drives the hemodynamic supply (HbO) in all E/N/M bands in the resting prefrontal cortex. Furthermore, to investigate the effect of transcranial photobiomodulation (tPBM), we performed a sham-controlled study by delivering an 800-nm laser beam to the left and right prefrontal cortex of the same participants. After performing the same data processing and statistical analysis, we obtained novel and important findings: tPBM delivered on either side of the prefrontal cortex triggered the alteration or reversal of directed network couplings among the three neurophysiological entities (i.e., HbO, CCO, and EEG frequency-specific powers) in the physiological network in the E and N bands, demonstrating that during the post-tPBM period, both metabolism and hemodynamic supply drive electrophysiological activity in directed network coupling of the prefrontal cortex (PFC). Overall, this study revealed that tPBM facilitates significant modulation of the directionality of neurophysiological networks in electrophysiological, metabolic, and hemodynamic activities.

## Introduction

### Physiological networks in the human prefrontal cortex

A new field of network physiology has formed for multidisciplinary research that investigates the dynamic states of not only anatomical or functional networks within an organ but also multiple coexisting forms of physiologic coupling^1,2^. The essence of this area of research is that a living human organism consists of a variety of physiological systems that interact within or among organs for normal operation and healthy conditions^2^. For instance, Hendrikx et al. developed a physiological network to analyze dynamic neurovascular coupling in neonates, while considering concurrently recorded oxygen saturation, carbon dioxide, and blood pressure in an integrative manner^3^. Following the same strategy, the current study aimed to construct and quantify a new set of physiological networks in the human prefrontal cortex among neuronal, vascular, and metabolic activities at rest and after noninvasive transcranial light stimulation.

Specifically, neuronal and vascular activities in the central nervous system (CNS) interact bidirectionally and have been observed in dynamic changes during their activities and referred to as neurovascular coupling (NVC)^4,5^. The mechanism of NVC is not fully understood, but it is believed that NVC is mediated by the hemodynamic supply that is linked to the metabolic and electrophysiological demands of oxygen and glucose.

From a vascular and metabolic perspective, spontaneous fluctuations in vasomotion are thought to play a key role in regulating metabolic and hemodynamic activities with three regulatory components of the CNS: (1) the endothelial layer of the cerebral vasculature and astrocyte glial cells as part of the blood–brain barrier (BBB), (2) neurons, and (3) smooth muscles on the blood vessel wall^3^. These components consist of three infra-slow oscillations (ISO)^6^ in the (1) endogenic (E; 0.005–0.02 Hz), (2) neurogenic (N; 0.02–0.04 Hz), and (3) myogenic (M; 0.04–0.2 Hz) frequency bands^7–9^. Currently available brain sensing and imaging modalities include functional magnetic resonance imaging (fMRI), functional near-infrared spectroscopy (fNIRS), fluorodeoxyglucose positron emission tomography (FDG-PET), and broadband fNIRS (bbNIRS)^10,11^, all of which facilitate noninvasive measurements of hemodynamic and/or metabolic activity in the human brain. Regarding electro-neurophysiological measurements, a multichannel electroencephalogram (EEG) is a portable, low-cost, noninvasive, brain-sensing technique with high temporal and spectral resolution. Therefore, it is commonly used in clinical settings and research studies. EEG signals are often analyzed in five frequency bands: delta (1–4 Hz), theta (4–8 Hz), alpha (8–13 Hz), beta (13–30 Hz), and gamma (> 30 Hz). Different EEG frequency bands have shown distinct coupling with hemodynamic activity in NVC studies based on the brain regions of interest, tasks, and neurological diseases^4,5, 12–15^.

The study of neurovascular, neurometabolic, and metabolic-vascular coupling in the human brain has recently garnered attention due to their pivotal roles in understanding cerebral functioning and potential implications in various neurological disorders. For instance, Shokri-Kojori et al. contributed to this discourse by elucidating the correspondence between cerebral glucose metabolism and BOLD signals through a multimodal investigation^16^. By directly linking metabolic demands with BOLD fluctuations, the authors shed light on the relative power and cost considerations within the human brain, offering a comprehensive view of the metabolic-vascular interplay. More recently, Pinti et al. presented an exemplary analysis framework for the integration of bbNIRS with EEG measurements and the concurrent quantification of neurovascular dynamics and neurometabolic activity^13^. This novel approach provides insights into the dynamic relationship between the local oxygen supply and neuronal energy consumption. Their findings underscore the importance of cross-modal analysis, revealing nuanced associations between neurovascular responses and the underlying neuronal processes.

### Noninvasive transcranial photobiomodulation (tPBM)

In recent years, tPBM has gained much attention and research interest as a non-invasive neuromodulation modality to enhance cognition in healthy humans and patients with neurological disorders^17–22^. The underlying principles and mechanistic actions of tPBM are comprehensively reviewed^17,23–25^. Accordingly, the most well-documented and accepted mechanism underlying the effects of tPBM is that complex IV, cytochrome *c* oxidase (CCO), in the mitochondrial respiratory chain can absorb near-infrared (NIR) light at the local stimulation site. Absorbed light stimulates cellular adenosine triphosphate (ATP) and releases nitric oxide (NO)^19^. Consequently, this signaling reaction boosts mitochondrial functions for human cognition in the normal brain and promotes energy supplementation in the disease-affected brain with prominent mitochondrial dysfunction^26^. Our group recently reported several noninvasive in vivo human studies to support the aforementioned mechanism of tPBM-induced excitation of mitochondrial CCO by showing significant increases in cerebral concentrations of oxidized cytochrome *c* oxidase (Δ[CCO]), oxygenated hemoglobin (Δ[HbO]), and total hemoglobin (Δ[HbT])^10,27–29^. However, studies on tPBM-stimulated electrophysiological responses in living human brains are limited, with only a few publications^30–33^. Furthermore, no article in the literature has reported tPBM-induced effects on neurophysiological networks.

### Aims of this study

Since this study aimed to assess cerebral coupling among different physiological activities of the prefrontal cortex at rest and under tPBM, we used a cross-modal brain-sensing technique by concurrently measuring 2-channel bbNIRS (2-bbNIRS) with 19-channel EEG. Such an approach enables noninvasive assessment of NVC, neurometabolic coupling (NMC), and metabolic-vascular coupling (MVC), all three of which facilitate to construct a physiological network in a specific prefrontal region. This network consists of three network nodes representing electrophysiological, hemodynamic, and metabolic activity at a local site. The coupling strength between each pair is determined by the coherence between the paired signals and represented for the respective network edge. The underlying rationale of this analysis is that the physiological coupling of two time series can be defined by a directed (i.e., effective) connectivity measure, where a strong or net unidirectional connectivity between two physiological signals implies a leading relationship from one physiological property to the other. Accordingly, this study aimed to prove the hypothesis that neurophysiological interactions in the resting human prefrontal cortex are directed among electrophysiological, hemodynamic, and metabolic activities at ISO frequencies, and that such directed connections are significantly altered/reversed by tPBM.

This paper serves as a ‘proof-of-concept’ study and reports three novel aspects of this study. First, we demonstrated concurrent measurements of 2-bbNIRS and EEG from the forehead of healthy young adults in vivo, in the resting state and after tPBM. Second, we introduced a novel formation of neurophysiological networks using generalized partial directed coherence analysis^34^, which enabled us to quantify and understand directed physiological networks in the prefrontal cortex at rest and after tPBM. Finally, the new findings of this study provide insights into the directed physiological networks of the NVC, NMC, and MVC in the human brain at rest and after tPBM.

## Materials and methods

### Human participants

A total of 31 healthy participants were initially recruited from the community of the University of Texas at Arlington^35^. They were screened using the same inclusion/exclusion criteria as those used in our previous studies^10,36, 37^. Namely, the inclusion criteria included: either sex, any ethnic background, and in an age range of 18–40 years old. The exclusion criteria included: (1) diagnosed before with a psychiatric disorder, (2) history of a neurological condition, or severe brain injury, or violent behavior, (3) have ever been institutionalized/imprisoned, (4) current intake of any medicine or drug, or (5) currently pregnant. In addition, none of the participants had diabetes. Nine subjects had excessive movement during the experiment and thus generated significantly noisy EEG data, so these participants were excluded from further data analysis. After exclusion, 22 young healthy humans (14 males and 8 females; age = 22.6 ± 2.7 years) participated in 5 visits of measurements separated by at least 7 days. In four out of five visits, each participant was randomly assigned to the left prefrontal 800-nm tPBM (L800), right prefrontal 800-nm tPBM (R800), left prefrontal sham (LS), or right prefrontal sham (RS) stimulation. The Institutional Review Board (IRB) of the University of Texas at Arlington approved all experimental procedures. All measurements were conducted with informed consent from each participant in accordance with the guidelines approved by the IRB of the University of Texas at Arlington.

To ensure that the number of participants was statistically adequate for the study, we performed a sample-size analysis based on an online calculator (https://clincalc.com/stats/samplesize.aspx) using the parameters obtained in our previous studies. Namely, a mean amplitude of Δ[HbO] oscillations in the range of 0.005–0.2 Hz was selected to be ~ 0.25 μM, as reported in Ref.^35^, with an estimated error of ~ 0.06 μM that was calculated using the error propagation method^38^. In addition, an expected tPBM-induced change in the amplitude of Δ[HbO] of ~ 20% with an alpha value of 0.05 and a power of 80% were the selected parameters used in the analysis, all of which resulted in a minimum sample size of ~ 23. Therefore, the participants matched the required minimal sample size after exclusion.

### Two-channel broadband NIRS and its setup

The utilization of 2-bbNIRS was recently introduced to quantify the prefrontal bilateral connectivity and unilateral coupling of Δ[HbO] and Δ[CCO] at the infraslow oscillations in the human brain at rest^35^ and post tPBM^28,39^. The working principle is based on the distinct light absorption of HbO, deoxygenated hemoglobin (HHb), and redox-state of CCO; their concentration changes or oscillations in living tissues can be quantified using the modified Beer–Lambert law^40–44^. A broadband spectroscopy measurement is required because of the lower concentration of CCO in comparison to HbO and HHb^11,38, 45, 46^. Concurrent determination of these changes permits investigation of the intertwined neurophysiological network between hemodynamic and metabolic ISO activities, which can be termed metabolicvascular coupling (MVC)^35,39^. Section A of the Supplementary Materials provides the mathematical principles and derivations for the quantification of Δ[CCO] and Δ[HbO]; Section B of the Supplementary Materials also offers a graphical demonstration of the decomposition of a Δ[HbO]/Δ[CCO] time series into E/N/M frequency bands.

**Figure 1:**
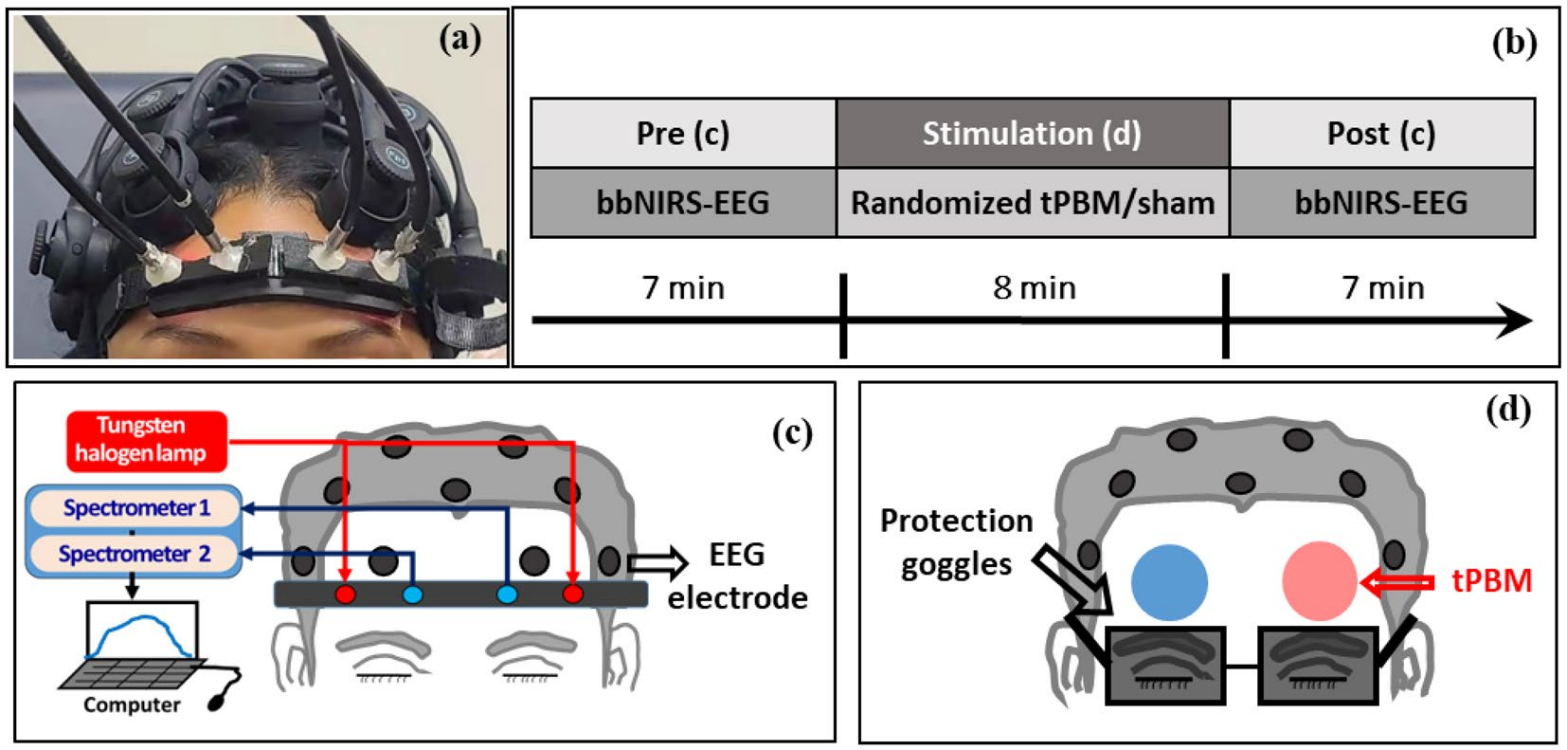
(**a**) Experiment setup including two channels of bbNIRS on the lateral forehead and EEG cap. (**b**) The experimental protocol of this study; it consists of 5 visits with 7-min eyes-closed pre-stimulation at rest, followed by 8 min of randomized tPBM or sham stimulation on the left forehead (L800), right forehead (R800), left sham (LS), or right sham (RS) in four of the five visits, and 7-min post-stimulation. (**c**) Schematic diagram illustrating an EEG and 2-bbNIRS setup; the latter consists of a light source for 2 channels of bbNIRS, two separate spectrometers, and optical connections. The 2-bbNIRS and EEG data were concurrently collected pre- and post-stimulation. (**d**) Locations of the tPBM/sham stimulation delivered on the left (red circle) or right (blue circle) forehead. The bbNIRS holder and EEG channel Fp1 or Fp2 were removed during the left or right tPBM stimulation.

The 2-bbNIRS system^35^ is briefly described below. As shown in Fig. 1a,c, the experimental setup consisted of two separate bbNIRS channels, which had a 3-cm source-detector separation and were placed on each subject’s forehead before and after tPBM was delivered to either side of the forehead. This 2-bbNIRS set was assembled with an EEG system (more details given below) to form a multimodal brain-sensing system to investigate neurophysiological networks in the human forehead (Fig. 1a,c). In principle, a longer integration time in bbNIRS data collection provides a better signal-to-noise ratio (SNR) but reduces the temporal resolution. Thus, we chose an integration time of 1.5 s (equivalent to a sampling rate of 0.67 Hz), which balances the SNR for bbNIRS and the temporal resolution well. With this sampling rate, the maximal frequency we can detect is 0.32 Hz, covering the ISO range of 0.005–0.2 Hz. More detailed information on this setup can be found in Refs.^28,35^.

### 19-Channel EEG device

EEG data were collected during the pre- and post-stimulation periods using a 19-channel, wireless, dry EEG device (Quick-20, CGX—Cognionics, CA, US), with dry electrode resistance between 10–100 kΩ for unprepared skin. As shown in Fig. 1, each participant wore an EEG cap according to the standard 10–20 EEG electrode placement^47^. The EEG time series were recorded at a sampling rate of 500 Hz and directed to a laptop computer via wireless transmission.

### Experimental protocols for data collection and tPBM

The experimental protocol for sham and tPBM conditions is shown in Fig. 1b. The total measurement time was 22 min, including a 7-min pre-stimulation (rest), an 8-min randomized tPBM/sham, and a 7-min post-stimulation period, similar to those used in previous studies^10,17^. The participants sat comfortably on a chair with their eyes closed during resting-state (or pre-stimulation) data collection (Fig. 1a or c). The bbNIRS holder and fibers were removed and the EEG channel Fp1 or Fp2 was displaced during the tPBM stimulation period (Fig. 1d); all EEG sensors were returned in place during the post-stimulation recording (Fig. 1a or c).

The device and dosage of tPBM used in this study were the same as those recently reported^28^, which quantified prefrontal responses to left 800-nm tPBM based on only 2-bbNIRS measurements without any analysis of EEG signals. In this study, we included four sets of measurement data from four visits with tPBM and sham deliveries on the left and right lateral sites on the forehead. The laser for 8-min tPBM was at 800 nm with a beam diameter of 42 mm and irradiance of 250 mW/cm^2^ to deliver light near the Fp1 or Fp2 location for the left or right tPBM, respectively. On the contrary, the irradiance of the laser was set 0.1 mW/cm^2^ with a black cap covering the laser aperture in the two sham experiments. The detailed conditions and setting parameters for the tPBM and sham experiments are listed in Table 1. This laser power is considered safe for light delivery to the human forehead because of following justifications. First, numerous human studies by several research groups reported that the 250 mW/cm^2^ laser irradiance facilitated positive and augmented effects on human cognition without any adverse or negative outcome in both healthy adults and patients with neurological diseases^17,18, 48^. Second, a computer-simulation model demonstrated that the thermal effect induced by a 500 mW/cm^2^ light irradiance on the human scalp would increase 0.25 C in the scalp and 0.04 C in the gray matter^49^. This finding was further supported by a magnetic resonance thermometry study of 20 human participants, in which a 318 mW/cm^2^ laser was induced and created no significant temperature differences between active and sham stimulation^50^. The underlying rationale is that only 1–2% of light energy can penetrate through the scalp and skull of the human head to reach the cortex^51^. Therefore, it has been used commonly in this area of research (including ours)^10,17, 27, 52^. For eye protection, each participant and experimenter wore a pair of protective goggles during the tPBM/sham stimulation.

**Table 1:** Laser stimulation parameters for active tPBM and sham.

Stimulation	Wavelength (nm)	Beam diameter (mm)	Power density (W/cm^2^)	Delivered total power at site (W)	Time (min)	Total dose (J)
tPBM	800	42	0.25	3.46	8	1662
Sham	–	42	0	0	8	0

In the following sections, for the left-forehead tPBM, we will call Channel 1 (or Channel 2) of the bbNIRS and Fp1 (or Fp2) EEG electrode ipsilateral sensors and Channel 1 (or Channel 2) of the bbNIRS and Fp2 (or Fp1) electrode contralateral sensors (see Fig. 1b). This convention will be followed for the right-forehead tPBM. In this way, we can clearly describe the detection locations with respect to the stimulation site or side.

### Overview of data processing steps

**Figure 2:**
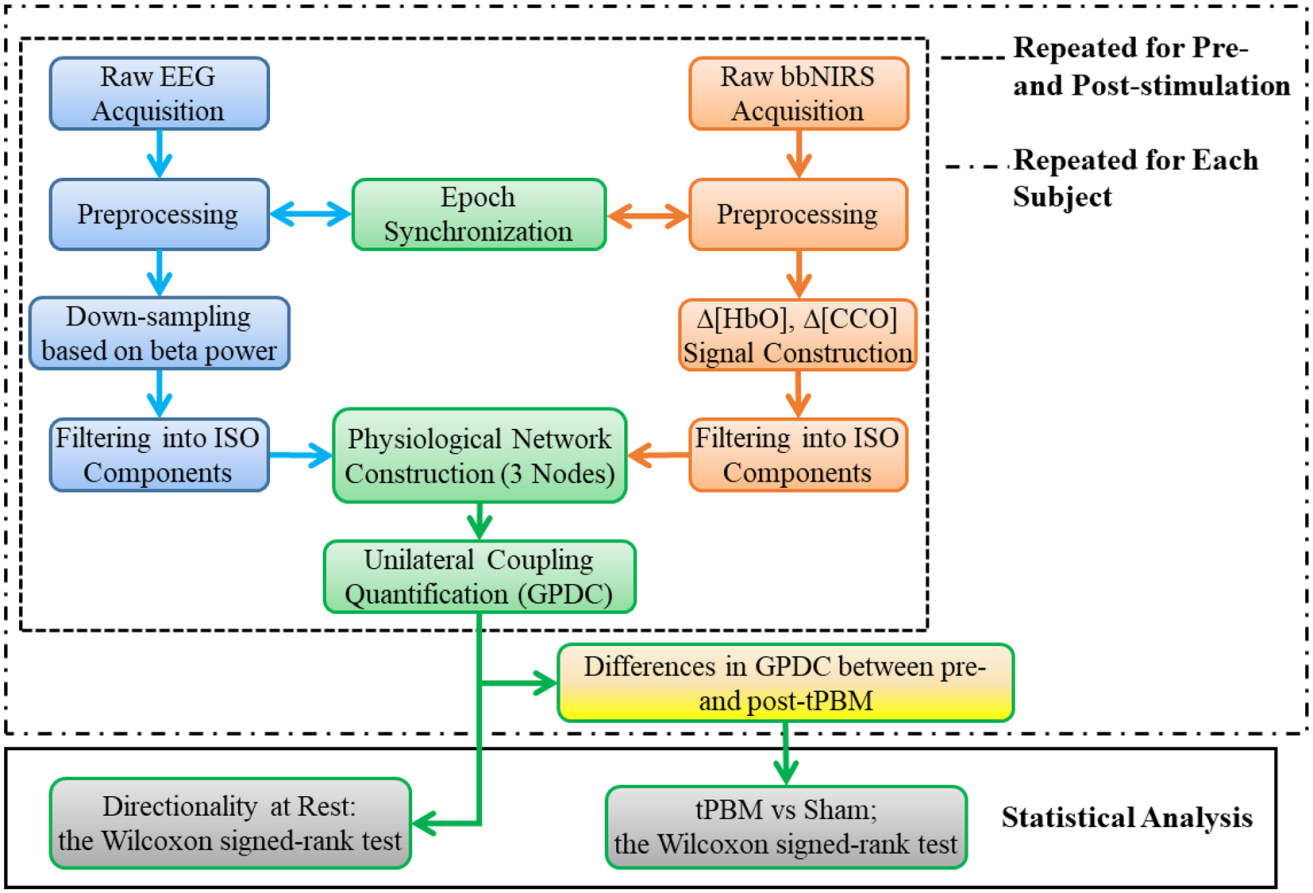
Data processing flow chart, including five steps for (1) EEG data analysis (blue boxes on the left of the figure), (2) 2-bbNIRS data analysis (orange boxes on the right of the figure), (3) construction of neurophysiological network and quantification of unilateral coupling using generalized partial directed coherence (GPDC) (green boxes), (4) quantification of differences in neurophysiological network between pre- and post-tPBM of respective GPDC values (as marked by the yellow box near the bottom of the figure), and (5) statistical analysis utilizing the Wilcoxon signed-rank test with FDR correction to assess the directionality in the neurophysiological network at rest and sham-controlled effects of tPBM (grey boxes on the bottom of the figure). All five steps were repeated for left and right forehead calculations. The dashed box outlines the repeated data-processing operation for the data collected before (i.e., at rest) and after tPBM/sham, while the dashed-dotted box outlines the repeated operation for each human participant.

After data acquisition, five processing steps were followed to construct and quantify neurophysiological networks based on 2-bbNIRS and EEG time series, as illustrated in Fig. 2. In brief, the five steps consist of (1) EEG data preprocessing and formation of down-sampled EEG power time series at each of alpha, beta, and gamma frequency bands for Fp1 and Fp2 EEG data, (2) 2-bbNIRS spectral data preprocessing and quantification of Δ[HbO] and Δ[CCO] time series, (3) neurophysiological network construction and unilateral coupling quantification using generalized partial directed coherence (GPDC), (4) calculations of differences in neurophysiological network between pre- and post-tPBM of respective GPDC values to quantify tPBM/sham-induced effect, and (5) statistical analysis to assess the directionality in the neurophysiological network at rest and sham-controlled effects of tPBM.

#### Step (1): EEG data preprocessing and formation of down-sampled time series of EEG powers

EEGLAB and FieldTrip, two open-source software toolboxes on the MATLAB platform, were used in conjunction to pre-process the EEG data. For preprocessing, each of the 19-channel raw EEG time series was band-pass filtered at 1–55 Hz using a Butterworth filter with an optimized order by a FieldTrip function, followed by re-referencing to the voltage averaged over all 19 channels. Next, independent component analysis (ICA)^53,54^ was used to remove noise and artifacts^55,56^ caused by eye movements, saccades, and jaw clenching. Then, artifact subspace reconstruction (ASR) was utilized to cover certain removed bad epochs using principal component analysis (PCA)^57^. Finally, clean EEG signals from electrodes Fp1 and Fp2 were selected to create their power spectral densities (PSD), which enabled us to calculate temporal variations of EEG powers in alpha, beta, and gamma frequency bands in the prefrontal area. The reason for selecting these three bands was that tPBM was reported by several studies to facilitate significant modulations of EEG powers in these frequency bands^30–33^**.**

**Figure 3:**
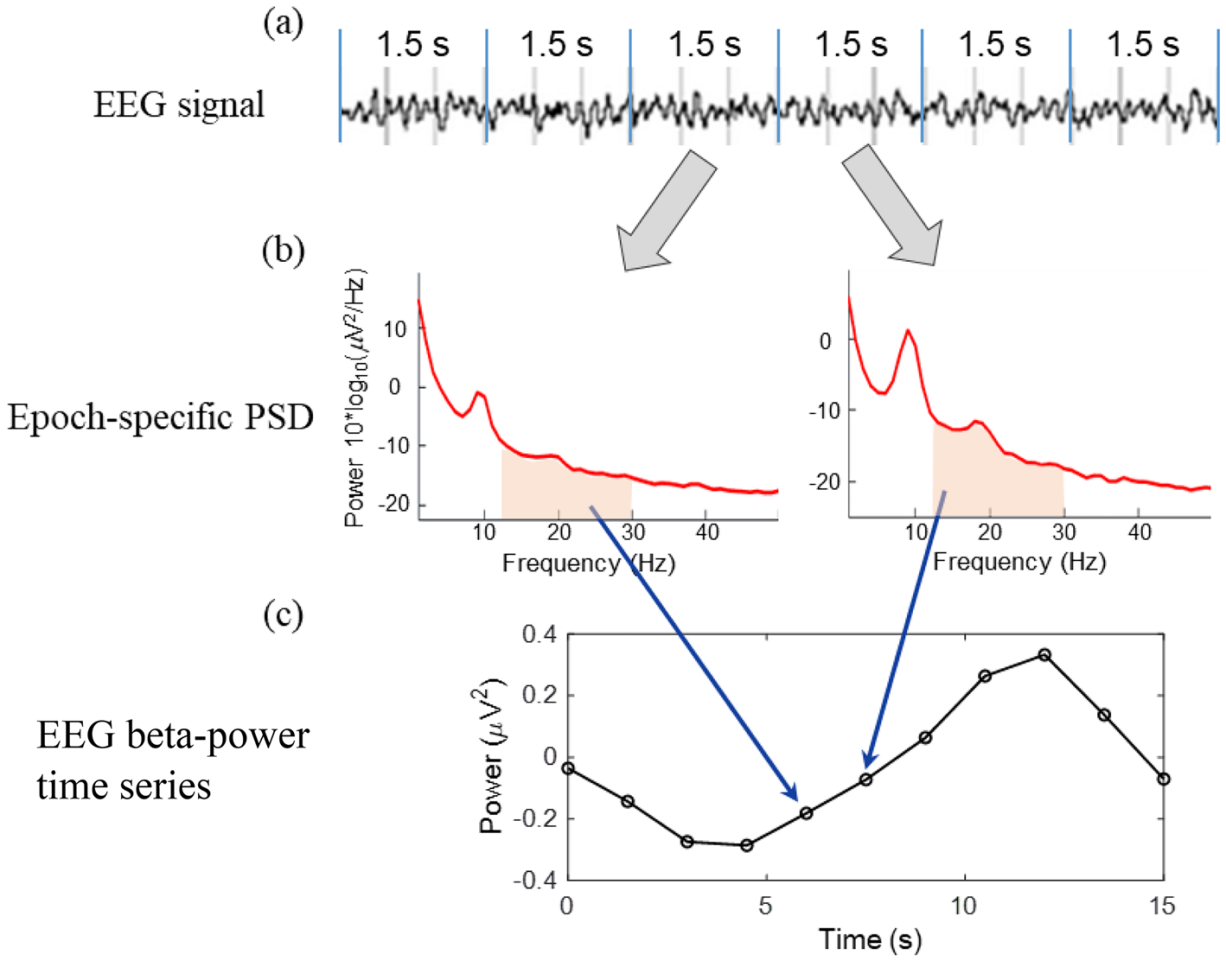
Schematic illustration of the process for down-sampling EEG power time series. (**a**) EEG time series segmented in 1.5-s epochs. (**b**) power spectral density (PSD) obtained from each epoch. As an example, the area under the curve in the beta band is calculated and used to construct (**c**) an EEG beta-power time series with a time resolution of 1.5 s, which is matched to that of 2-bbNIRS.

To quantify the coherence of two time series, EEG data had to be down-sampled from 500 Hz to match the sampling rate of 2-bbNIRS (0.67 Hz), following the method described in^13^. Figure 3 schematically illustrates the steps of the down-sampling process. Specifically, the EEG data were segmented into 1.5-s epochs (i.e., the sampling rate of 2-bbNIRS), and the power spectrum density (PSD) of the EEG signal was obtained for each epoch using a single-tapered Fast Fourier Transform (FFT). The area under the curve of PSD for each of alpha (8–13 Hz), beta (13–30 Hz), and gamma (30–55 Hz) band was calculated and used to construct a time series of EEG alpha, beta, and gamma power with 1.5-s temporal resolution (0.67 Hz, 280 time points).

#### Step (2): bbNIRS data preprocessing

The 7-min bbNIRS dataset with a sampling rate of 0.67-Hz (i.e., 1.5 s per data point) included 280 temporal points, each of which contained a backscattered NIR spectrum with a spectral interval of 0.38 nm. The wavelength range of 780–900 nm was used to estimate changes in concentration of HbO (i.e., Δ[HbO]) and CCO (i.e., Δ[CCO]) using the modified Beer–Lambert law in each time point^11,46, 58, 59^. The time series of Δ[HbO] and Δ[CCO] were then obtained and filtered into three ISO frequency bands (i.e., 0.005–0.2 Hz). Section A of the Supplementary Materials outlines the mathematical principles and steps for the quantification of Δ[CCO] and Δ[HbO], and Section B of the Supplementary Materials provides a graphical demonstration of the decomposition of a Δ[HbO] time series into E/N/M frequency bands. The effect of motion and systemic artifacts on ISO was minimal since such signals were usually in higher frequencies. Also the data from subjects who had excessive motion were excluded for group analysis. To reduce motion artifacts, medical double-sided tapes were applied on the probe–skin interface to further secure the probe on the head and minimize motion artifacts.

#### Step (3): Algorithm for construction of unilateral neurophysiological network

Physiological coupling of two or more time-series can be quantified by directed (effective) connectivity measures where strong unidirectional connectivity between two signals implies an effective relationship from one signal to the other. Bivariate and multivariate algorithms can be used to reveal the effective connectivity between nodes in a network. Bivariate measures of connectivity, such as cross-correlation, coherence, and Granger causality (GC), can be used to determine the connectivity between an isolated pair of signals from a network at a time. On the other hand, multivariate measures, such as directed transfer function (DTF) and partial directed coherence (PDC), can account for the entire multivariate structure of a network using a multivariate autoregressive model (MVAR). However, because of the reported shortcomings and pitfalls of each method^34,60^, more robust methods have been developed. For instance, generalized partial directed coherence (GPDC) is a modified version of the PDC and focuses on sources rather than sinks in directed connectivity^34^. More detailed comparison between PDC and GPDC is provided in Section C of the Supplementary Materials.

In this study, we first took MVAR that enabled us to consider all the links (i.e., interactions) between all nodes in a network simultaneously. Specifically, for a *k*-node process of X(t), we have:

1 $$X\;(t) = ({X_{1}} (t),\;{X_{2}} (t), \; \ldots , \;{X_{k}} (t)),$$

where *X*_*k*_(*t*) expresses a time series of the *kth* physiological signal. The MVAR model of *X*(*t*) can be represented as:

2 $${\varvec{X}}\left(t\right)=\sum_{n=1}^{p}{\varvec{A}}\left(n\right){\varvec{X}}(t-n){\varvec{E}}\left(t\right),$$

where ***A*** is the *k* × *k*-sized matrix of coefficients, ***E***(*t*) is a prediction error vector of size *k*, and *p* is the order of the model. The MVAR model can be transformed to the frequency domain (see Section C of the Supplementary Materials for details). After a few steps of further processing, the generalized partial directed coherence (GPDC) at frequency *f* then can be expressed as^61^:

3 $$GPDC_{j,i} (f) = \frac{{\frac{1}{{\sigma_{i} }}A_{ij} (f)}}{{\sqrt {\sum\nolimits_{n = 1}^{k} {\frac{1}{{\sigma_{n}^{2} }}A_{nj} (f)A_{nj}^{*} (f)} } }},$$

where *A*_*ij*_(*f*) is an element of *A*(*f*)—a Fourier transform of MVAR model coefficients *A*(*t*)^34^, the asterisk denotes the complex conjugate transpose, and $\sigma_{n}^{2}$ refers to the variance of the prediction error of the *n*th time series (i.e., *E*_*n*_(*t*))^61^. In this study, *k* is equal to 3, representing the three neurophysiological metrics. Thus, the output adjacency matrix is a 3 × 3-sized nonsymmetrical matrix with elements of GPDC_*j,i*_*,* where the element in column *j* and row *i* denotes the effective (directed) connectivity from node *j* to node *i*.

#### Step (4): Physiological directionality assessment at rest and after tPBM

To investigate the causal interaction between different physiological representations of local brain activity (i.e., the left or right prefrontal cortex) in the resting state, hemodynamic, metabolic, and electrophysiological signals taken at rest were integrated to construct a set of 3-node neurophysiological networks. This set of networks consisted of three nodes, Δ[HbO], Δ[CCO], and EEG powers at different frequency bands, and three edges/links of NVC, NMC, and MVC. To obtain the links connecting these nodes, GPDC was calculated for all 3 × 3 possible directed connections, and the neurophysiological network was constructed at each ISO frequency band, as expressed ${\text{GPDC}}_{j,i}^{f}$, where *f* denotes the three E/N/M bands, for the resting prefrontal cortex. In this study, we set the order of the MVAR model to be 1 based on the Akaike information criterion (AIC), a method to evaluate the goodness of fit, and similar studies investigating ISO hemodynamic connectivity^62,63^.

Furthermore, the interest of this study included a reliable observation of tPBM effects on the prefrontal neurophysiological network compared with the resting state. It is meaningful to determine changes of ${\text{GPDC}}_{j,i}^{f}$ between pre- and post-stimulation of a human subject for each pair of neurophysiological network links (i.e., NVC, MVC, and NMC). This change can be expressed mathematically as follows:

4 $$\Delta GPDC_{j,i}^{f} = GPDC_{j,i,post}^{f} {-}{\text{ GPDC}}_{j,i,pre}^{f} ,$$

where *j* and *i* cover Δ[HbO], Δ[CCO], and EEG frequency-specific powers (i.e., alpha, beta, and gamma bands). Indeed, Eq. (4) expresses a within-subject difference (i.e., between pre- and post-stimulation) and can be applied to each of the tPBM and sham experiments. Therefore, to minimize the placebo effect and enhance detection sensitivity, sham-subtracted changes by tPBM in neurophysiological unilateral coupling were written as follows:

5 $$\Delta GPDC_{j,i,ss}^{f} = \Delta GPDC_{j,i,tPBM}^{f} {-}\Delta {\text{GPDC}}_{j,i,sham}^{f}$$

These values were then utilized to illustrate the difference in physiological networks between sham and active tPBM conditions.

#### Step (5): Statistical analysis to assess the directionality in the neurophysiological network

To conduct an appropriate statistical analysis, we ran a normality test on each of the calculated directionalities in the neurophysiological network at rest (i.e., pre-tPBM) and post-tPBM. However, some directionalities are not normally distributed. Thus, the Wilcoxon signed-rank test, as a non-parametric hypothesis test, was used for statistical analysis in this study. This test was chosen because it is typically applied when the data are paired or matched.

Specifically, two types of statistical analysis were performed. First, for the resting-state prefrontal cortex, the Wilcoxon signed-rank test was computed for each pair of GPDC ^*f*^_*j,i*_ variables for the three links (i.e., NVC, MVC, and NMC) at each of the E/N/M bands for each case with the EEG alpha, beta, and gamma powers. This analysis was repeated for the left and right prefrontal cortices (PFC). A significance level of p < 0.05 was set with FDR correction for multiple comparisons. This operation enabled us to identify significant directed/effective connectivity from one physiological network node to another in the resting prefrontal cortex. Second, for tPBM-induced effects, the respective pre-stimulation-subtracted coherence adjacency matrices of $\Delta GPDC_{j,i}^{f}$ (Eq. (4)) were determined for each unidirectional connection strength for both tPBM and sham treatments. The Wilcoxon signed-rank test between Δ*GPDC*_*j,i,tPBM*_ and Δ*GPDC*_*j,i,sham*_ for each respective pair was again performed with a significance level of p < 0.05 (FDR corrected). After the statistical testing, only significant unilateral connections (p < 0.05) were reported in the network illustration with sham-subtracted Δ*GPDC*_*j,i,ss*_ values (Eq. (5)). This statistical operation was repeated for both the L800 and R800 tPBM.

### Ethical approval and informed consent

All research and methods were carried out in accordance with relevant guidelines and regulations. The Institutional Review Board of the University of Texas at Arlington approved all experimental protocols. All participants provided written informed consent in accordance with the guidelines approved by the Institutional Review Board of the University of Texas at Arlington.

## Results

### Unilateral neurophysiological networks of the PFC in the resting human brain

After preprocessing of the dual-mode data taken in the resting state, three different time series of neurophysiological signals, namely, Δ[HbO], Δ[CCO], and down-sampled EEG power at a selected frequency (i.e., alpha, beta, or gamma) band were used to construct resting-state neurophysiological networks over the left or right PFC. These three-node networks were constructed individually for each of the three ISO bands (E/N/M) using the MVAR model, followed by GPDC. Specifically, a constructed adjacency matrix consists of three nodes labelled by HbO, CCO, and EEG (for a selected frequency band) for simplicity. The calculated GPDC values between each pair of nodes provided two directed connectivity and represented their respective coherence strengths.

**Figure 4:**
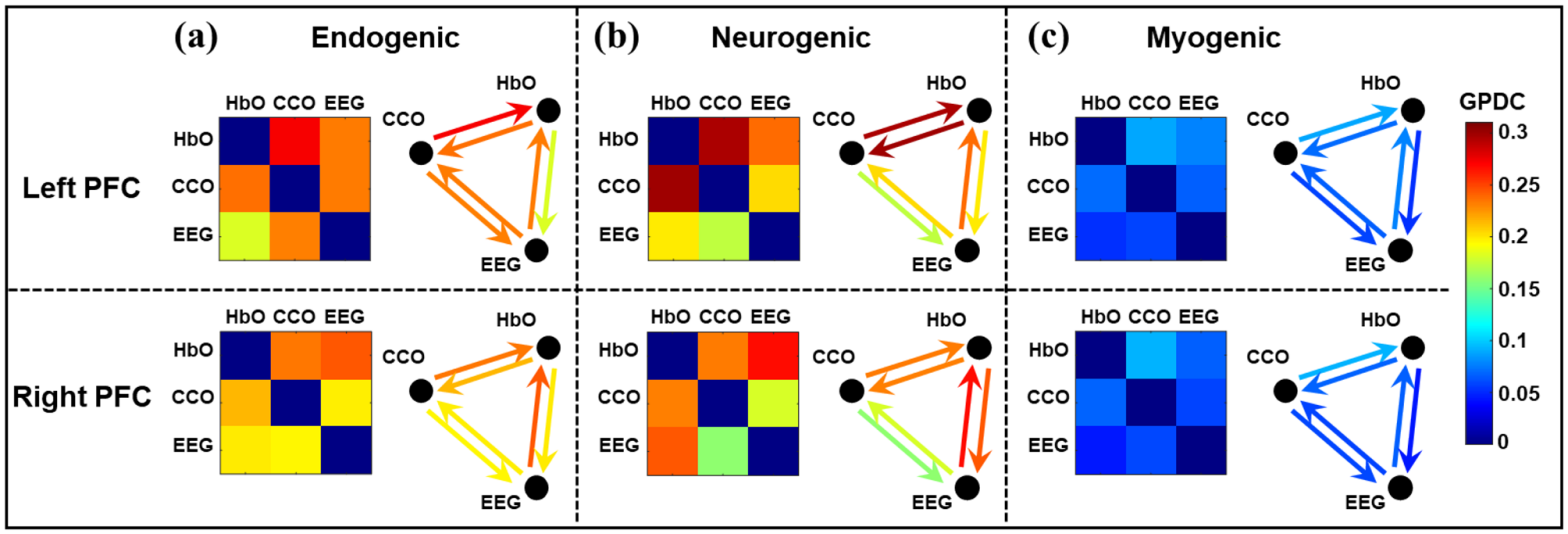
Adjacency matrices and graphical illustration of resting-state neurophysiological networks on the prefrontal cortex obtained from dual-mode 2-bbNIRS and EEG dataset. Three columns represent (**a**) endogenic, (**b**) neurogenic, and (**c**) myogenic components of ISO over left and right PFC. The nodes in each network are HbO, CCO, and EEG beta power. (n = 22; data were averaged over 5 repeated measurements).

Because the GPDC calculation for coherence with EEG beta power would be the same with EEG alpha and gamma powers, we first show the GPDC results using EEG beta power as the electrophysiological node. Figure 4 represents the adjacency matrices and graphical illustration of the three resting-state neurophysiological networks over the left and right PFC in the E/N/M bands. As illustrated in Fig. 4a, all three (NVC, MVC, and NMC) coupling strengths were relatively high in the endogenic band, indicating a closely coupled neurophysiological activity among the three nodes of the network on both lateral PFC. Figure 4b also shows strong coupling between CCO-HbO (i.e. MVC) and EEG-HbO (i.e. NVC) in the neurogenic oscillation of ISO, whereas CCO-EEG (NMC) coupling is comparably weak in this frequency band over both lateral PFC. In comparison, all three couplings were much weaker in the myogenic band in both lateral PFC.

**Figure 5:**
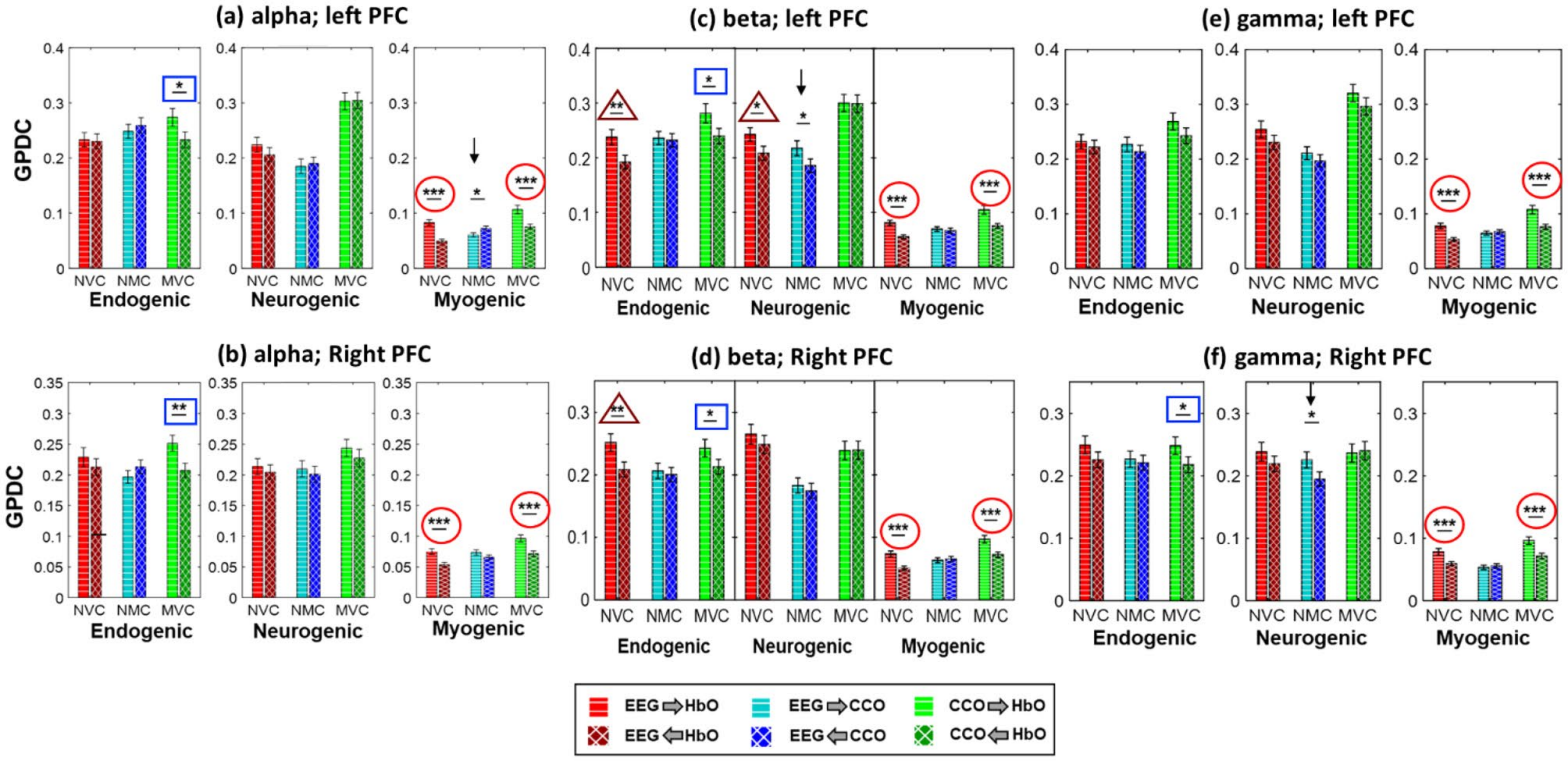
Directionality assessment in the neurophysiological network links, grouped by coherence with EEG (**a**,**b**) alpha power, (**c**,**d**) beta power, and (**e**,**f**) gamma power. The top and bottom rows correspond to the network links of the left and right PFC at rest. *NVC:* neurovascular coupling, *NMC:* neurometabolic coupling, *MVC:* metabolicvascular coupling. *p < 0.05, **p < 0.01, ***p < 0.001 obtained from the Wilcoxon signed-rank test after FDR correction (n = 22; data were averaged over 5 repeated measurements). Red circles mark highly significant leading roles of EEG and CCO with respect to HbO in the M band, consistently over both lateral PFC and across all three EEG powers. Blue rectangles make significant leading roles of CCO compared to HbO in the E band, consistently over both PFCs for EEG alpha and beta powers. Three triangles mark significant leading roles of EEG compared to HbO only in the EEG beta power case. Two downward arrows mark significant leading roles of EEG beta and gamma activities compared to CCO in the N band of the left and right PFCs, respectively.

The directionality of each link in each neurophysiological network, including NVC, NMC, and MVC at rest, reveals the possible leading role of one neurophysiological entity or element to another. Thus, to statistically assess the difference between two opposite connectivity directions, the Wilcoxon signed-rank test was performed for each link for each of the three networks. For the directed coherence with the EEG beta power, the results are shown in Fig. 5c,d, respectively, for the left and right PFC. These two panels illustrate that, in the case of NVC (red bars), the directional coupling of EEG to HbO (EEG → HbO) is significantly larger than that of HbO → EEG in both the E and M bands on both lateral sides and in the N band on the left PFC. A similar unidirectional pattern was also evident in MVC (green bars) in both E and M bands; namely, the directional coupling of CCO → HbO was stronger than that of HbO → CCO in these two bands. This set of observations implies that oscillations of both EEG beta power and cerebral CCO drive that of cerebral HbO over the E and M bands on both lateral sides. However, for NMC (i.e., CCO vs. EEG), few significant difference between the two opposite directional coupling was observed.

Following the same analysis steps, we obtained statistical comparisons of significant directed coherences for each link in each neurophysiological network using EEG alpha and gamma powers, as shown in Figs. 5(a,b) and Fig. 5(e,f), respectively. A close inspection on Fig. 5 reveals several key observations: (1) In the myogenic band, both EEG powers and CCO activities led or drove HbO consistently over both lateral PFC and across all three EEG powers (marked by red circles). (2) In the endogenic band, CCO activity led HbO consistently over both PFCs for EEG alpha and beta powers plus for EEG gamma power on the right PFC (marked by blue rectangles). (3) In the neurogenic band, only two cases showed significant leading roles of EEG beta and gamma activities compared to CCO on the left and right PFCs, respectively (marked by two black downward arrows). (4) In both the endogenic and neurogenic bands, the EEG beta power exhibited significant leading roles compared to HbO (marked by dark red triangles).

### tPBM-induced alterations in unilateral neurophysiological networks

**Figure 6:**
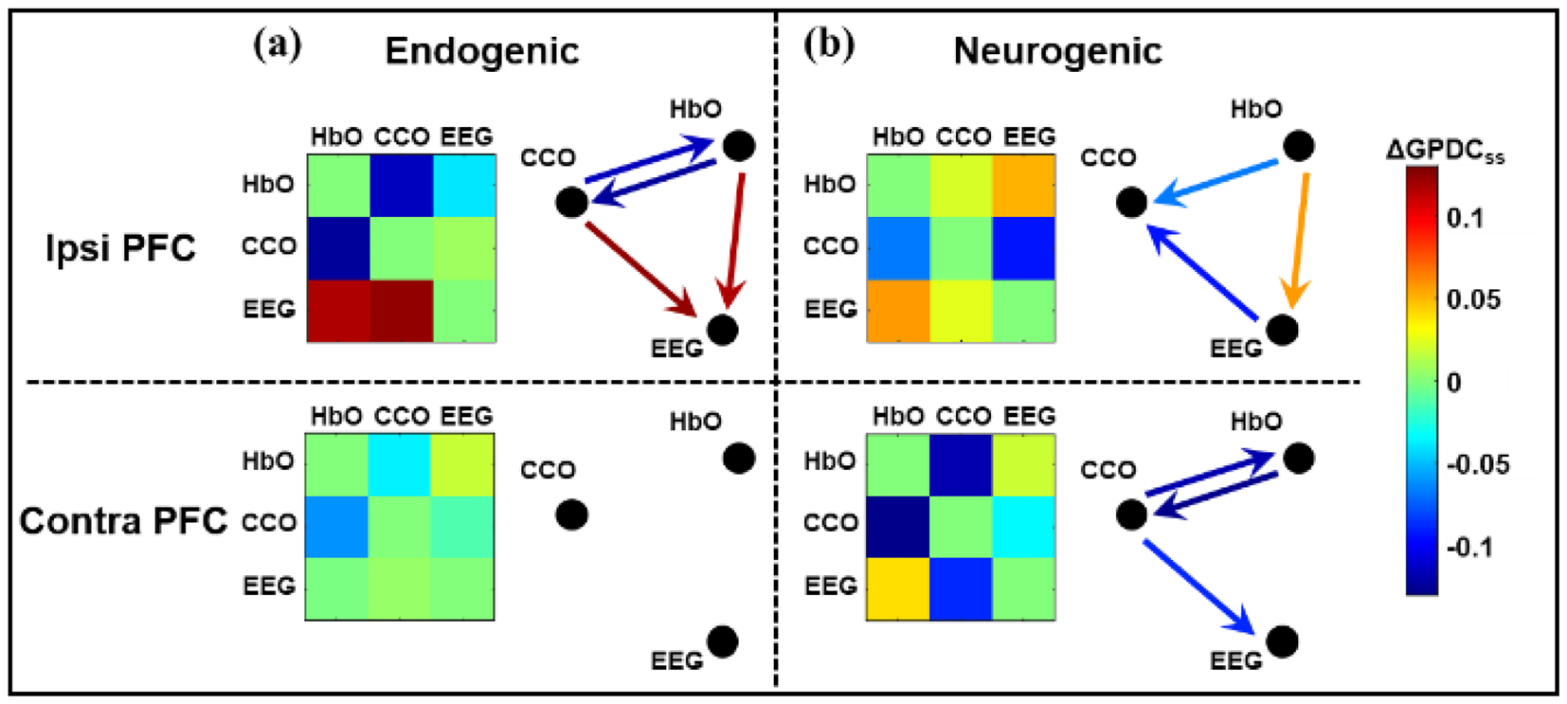
Adjacency matrices and graphical illustration of changes (i.e., ΔGPDC_ss_) in unilateral coupling in neurophysiological networks on the prefrontal cortex in response to L800 tPBM, as an example. Note that EEG beta power was considered in this case. Two columns represent (**a**) endogenic and (**b**) neurogenic components of ISO over the ipsi and contra PFC. The nodes in the network are HbO, CCO, and EEG beta power. Drawn links are the ΔGPDC_*j,i,ss*_ values, for which the Wilcoxon signed-rank test between ΔGPDC_j,i,tPBM_ and ΔGPDC_j,i,sham_ had to pass a p-value threshold of less than 0.05 (FDR corrected for multiple comparisons) (n = 22).

Following Eq. (5), the sham-subtracted values of ΔGPDC (i.e., $\Delta {\text{GPDC}}_{j,i,ss}^{f}$) for each directional coupling pair were calculated and statistically tested using the Wilcoxon signed-rank test for their significant alterations by tPBM. Significant changes in directed coherence by tPBM are plotted as color-coded adjacency matrices in Fig. 6. In this case, L800 was delivered and only EEG beta power was considered for the GPDC analysis. Since no significant change in directed coupling or coherence was identified in the myogenic band, only $\Delta {\text{GPDC}}_{j,i,ss}^{f}$ values in endogenic and neurogenic bands are reported in this figure. In addition to adjacency matrices, a graphical representation of the network is provided to help visualize changes in the directed network by reporting color-coded statistically significant links. Note that the terms of “ipsi PFC” and “contra PFC” indicate the tPBM and constructed network on the same and opposite PFC sides, respectively.

On the ipsi PFC (i.e., the same side as tPBM), Fig. 6a shows that in the endogenic band, tPBM resulted in a significant increase in directed coupling of both HbO → EEG and CCO → EEG marked by the dark red arrows, while bidirectional couplings between HbO and CCO significantly decreased as indicated by the blue arrows. In addition, in the neurogenic band, unidirectional coupling from HbO to EEG (orange arrows in Fig. 6b) was significantly increased, whereas the leading role of EEG and HbO with respect to CCO was significantly reduced on the same side (blue arrows in Fig. 6b). On the contra PFC, significant decoupling between CCO and HbO and a decrease in effective coupling from CCO to EEG were observed only in the neurogenic band.

**Figure 7:**
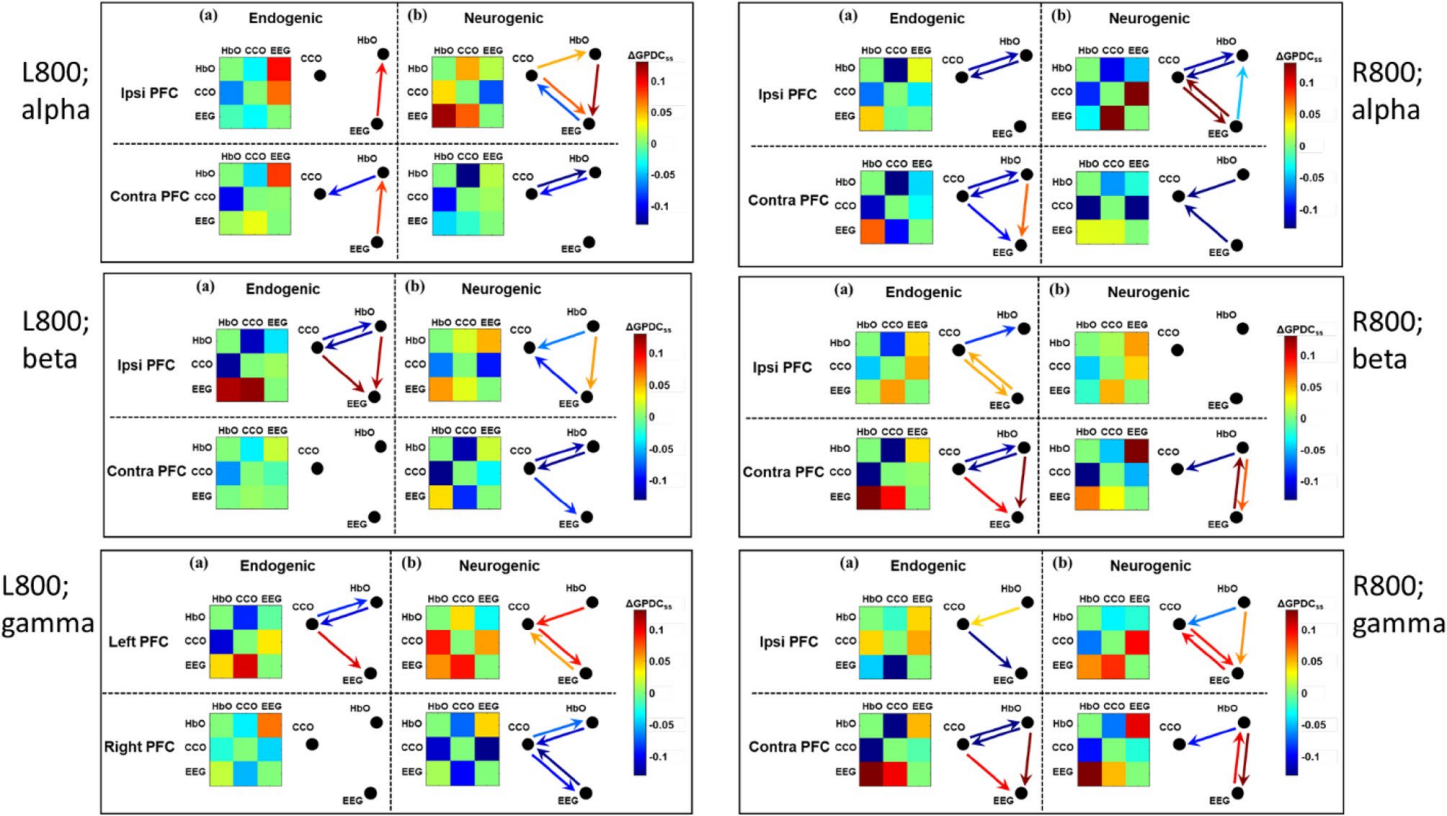
Adjacency matrices and graphical illustration of tPBM-induced significant changes in unilateral coupling in neurophysiological networks on the PFC in response to R800 tPBM (left column) and L800 (right column). Drawn links are the ΔGPDC_*j,i,ss*_ values (scaled by the color bars), for which the Wilcoxon signed-rank test between ΔGPDC_*j,i,tPBM*_ and ΔGPDC_*j,i,sham*_ passed p < 0.05 (with FDR correction). Note that labels of “alpha”, “beta”, and “gamma” in three respective rows denote EEG alpha, beta, and gamma powers used for GPDC calculations. The nodes in each network are HbO, CCO, and EEG power at a selected band.

Following the same analysis approach and presentation format used for Fig. 6, we obtained adjacency matrices and graphical drawings for tPBM-induced significant changes in unilateral coupling with EEG alpha and gamma powers for both the R800 and L800. A complete set of such matrices and graphical drawings is shown in Fig. 7. Specifically, the left column displays tPBM-induced changes by R800 in the (a) endogenic and (b) neurogenic bands when EEG alpha, beta, and gamma powers were considered for coupling. Similarly, the right column presents the changes caused by L800 with the same plot labelling and style. In the meantime, unidirectional links next to each network matrix in either endogenic or neurogenic frequency are color-coded for their values of ΔGPDC_*j,i,ss*_ and mark significant changes in directed connectivity with respect to the sham treatment. This comprehensive figure demonstrates that either R800 or L800 stimulated many significant alterations in effective/directed neurophysiological networks in both ipsilateral and contralateral PFC. To obtain meaningful results more easily and clearly, we made Table 2 to summarize the trends of network changes in coupling strength or/and directionality in the PFC induced by either R800 or L800.

**Table 2:**
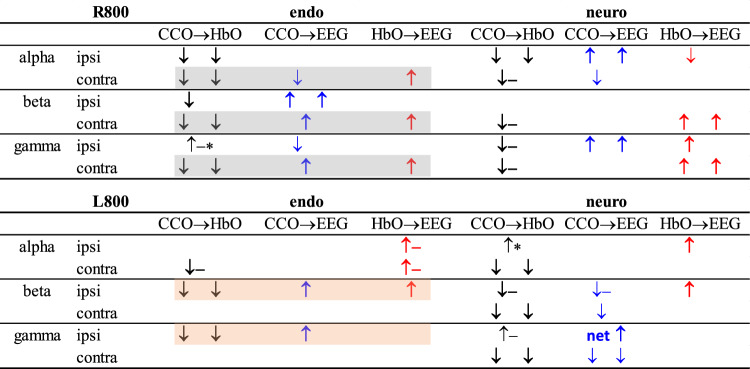
Trends of changes in network coupling in PFC induced by R800 or L800. Note that each arrow in the table represents a corresponding arrow in Fig. 7 under respective conditions.

“↓” and “↑” indicate a decrease and increase of coupling for each respective pairs.

Bolded black “**↓**” highlights the consistent trend of directional decoupling or reduction of CCO → HbO.

Bolded red “**↑**” highlights the consistency of increases in directional coupling of HbO → EEG.

Bolded blue “**↑**” highlights the consistency of increases in directional coupling of CCO → EEG.

“–” denotes an opposite directionality to the directional coupling given in the column label.

“*” indicates a marginal significance.

“ipsi” and “contra” mean the ipsilateral and contralateral PFC with respect to the tPBM side.

“endo” and “neuro”mean endogenic and neurogenic frequencies of ISO.

“alpha,” “beta,” and “gamma” mean EEG alpha, EEG beta, and EEG gamma powers.

Table 2 presents several observations. First, tPBM with L800 or R800 enabled significant decoupling or reduction of the directional connectivity between CCO and HbO (bold black arrows) compared to the sham intervention. This trend was rather global and consistent across the three EEG powers (alpha, beta, and gamma), in the ipsilateral and contralateral PFC, and in either endogenic or neurogenic bands. Second, both L800 and R800 also stimulated relatively global increases in the directional connectivity of HbO → EEG (bold red arrows) and CCO → EEG (bold blue arrows) across the three EEG powers in both the lateral PFCs and two ISO rhythm bands.

Furthermore, regional differences created by L800 and R800 in the directional connectivity of neurophysiological networks are evident: (1) In the endogenic rhythm, R800 created similar and significant network alterations on the contralateral PFC for all three EE powers (gray-shaded blocks). This pattern of change holds well in the ipsilateral PFC for EE beta and gamma powers by L800 (orange-shaded blocks). Taken together, these results imply that either L800 or R800 modulates more directional connectivity in the left PFC, particularly directional couplings with EEG beta and gamma powers, leading to decoupling of CCO → HbO and increases in the coupling of CCO → EEG. (2) In the neurogenic band, R800 increased the bidirectional connectivity of HbO → EEG with EEG beta and gamma powers in the contralateral PFC, and L800 augmented a similar trend for EEG alpha and beta powers in the ipsilateral PFC. Again, this set of results showed that the left PFC was a favored location, receiving or benefitting from more modulation of directional connectivity in neurophysiological networks from both L800 and R800.

## Discussion

Figure 4 illustrates three resting-state neurophysiological networks constructed among hemodynamic, metabolic, and electrophysiological activity at three ISO frequencies on the bilateral sides of the PFC derived from 110 measurements of 22 young healthy adults. Specifically, the links in each neurophysiological network in each ISO frequency band over each lateral (left and right) PFC consist of directed (1) unilateral neurovascular coupling (NVC), (2) unilateral neurometabolic coupling (NMC), and (3) unilateral metabolic-vascular coupling (MVC). Because the EEG time series includes a broad frequency band, we quantified electrophysiological activities mainly in three distinct alpha, beta, and gamma bands separately. These three bands were chosen because they are associated with wakeful, conscious, alert, and high-level cognitive brain operations or states compared to delta and theta rhythms. In the following subsections, we discuss the directed physiological couplings for each pair of links in the PFC at rest and after tPBM.

### Significant directed network links of EEG → HbO and CCO → HbO in the myogenic band of the rest PFC

All six panels in Fig. 5 show positive directional connectivity in the NVC (i.e., EEG → HbO) and MVC (i.e., CCO → HbO) in the myogenic band (marked by red circles), implying that alterations in electrophysiological and metabolic demands drive the hemodynamic supply in the neurophysiological network. This observation is remarkably consistent over both the left and right PFC, and across all three EEG frequency-specific powers in this band. It is also noted that the average strength of the directed coupling in this band was much lower than those in the other two ISO bands, as shown in Figs. 4c and 5. This phenomenon may be attributed to the fact that myogenic oscillations are highly associated with smooth muscle contraction–relaxation cycles. Thus, the demand and supply relationship in this frequency band is relatively more stable and weaker than those in the other two bands, independent of the local electrophysiological (alpha, beta, and gamma) rhythm frequencies and the two lateral sides of the resting PFC. Furthermore, the observed network directionality of EEG → HbO and CCO → HbO in the resting PFC is expected because both active electrophysiological and metabolic activities demand an essential oxygen supply offered through HbO. All these interpretations are supportive by recent publications^64,65^, stating that local neural activation drives an increase of regional blood flow to quickly supply oxygen and nutrients.

### Significant directed network links of EEG → HbO and CCO → HbO in the endogenic band of the rest PFC

In the endogenic band, CCO activity showed a significant leading role in directed connectivity to HbO (i.e., CCO → HbO) consistently over both lateral sides of the PFC when either EEG alpha or beta power was considered a node of the neurophysiological network (marked by blue rectangles in Fig. 5). A similar statement holds for the right PFC when the EEG gamma power served as a node of the network. This observation is in good agreement with the neurophysiological source of oscillation in the endogenic band where the contraction and relaxation of the endothelial layer of the vessel wall are a key component of the blood–brain barrier (BBB), which regulates the hemodynamics, metabolism, and neural activity in the human brain^12,66^. In other words, the oxygen demand–supply balance between cerebral hemodynamics versus metabolic activity is mainly controlled in this frequency band. Thus, changes in the BBB permeability (i.e., oxygen supply) are closely coupled and driven by metabolism and neural activity (i.e., oxygen demand) in the resting brain. Accordingly, the oxygen demand from neurological and metabolic activity leads or drives the oxygen supply offered by HbO in this frequency band. Note that the coupling strengths of MVC (CCO → HbO) in the endogenic band were approximately 2–2.5 times stronger than those in the myogenic rhythm (comparing those marked by rectangles and by red circles in Fig. 5).

Furthermore, when EEG beta power was served a node of the physiological network, we clearly observed significant directed NVC (i.e., EEG → HbO) in the endogenic band for both lateral PFC and in neurogenic band on the left PFC (marked by dark red triangles in Fig. 5). Such coupling strengths appear to be also much higher than those in the myogenic band. These observations confirmed again a directed information flow from electrophysiological activity to the highly coupled hemodynamics, as expected from the physiological definition of endogenic and neurogenic bands^67^.

### Significant directed network links of EEG → CCO in the neurogenic band of the rest PFC

In the neurogenic band, we observed only two significant directed links of NMC, with EEG beta and gamma powers leading to CCO metabolism (i.e., EEG → CCO) in the left and right side of the PFC, respectively (marked by two black downwards arrows). Overall, this result shows the most inconclusive and fewest net links observed across all six panels in Fig. 5. This implies that in the resting state, the directional or net coupling between electrophysiological and mitochondrial activities is much weaker and inconsistent, compared to the directed network links of EEG → HbO and CCO → HbO, across both lateral sides of the PFC in three ISO frequencies and with different EEG frequency powers.

### tPBM-induced reversal of EEG → HbO and CCO → HbO in physiological networks of the resting PFC

Significant alterations in the sham-subtracted directional connectivity in the neurophysiological network (i.e., ΔGPDC_ss_, as given in Eq. (5)) are illustrated in Fig. 7 for the ipsilateral and contralateral sides of the PFC under either R800 or L800 tPBM. Two key observations of the trends of changes in network coupling, as listed in Table 2, reveal interesting and vital findings.

First, R800 tPBM enabled a significant reduction in coupling in CCO ↔ HbO and a significant increase in coupling of CCO → EEG and HbO → EEG compared with those under the sham intervention. Specifically, both the reduction and increase were consistent or global in the contralateral PFC in the endogenic band, regardless of which EEG frequency-specific power served as a node of the network (see the three gray boxes in the upper-left portion of the table). In the neurogenic band, common features showing a significant reduction in HbO → CCO (equivalent to an increase in CCO → HbO) and significant increases in CCO → EEG and HbO → EEG were present clearly on either or both lateral sides of the PFC, depending on which EEG rhythm power was considered as a node in the physiological network. These observations are supported by previous reports^29,39^, suggesting that tPBM independently stimulates both hemodynamic (HbO) and metabolic (CCO) activities. The former may result from tPBM-induced release of nitric oxide (NO), which leads to alterations in contraction–dilation of the endothelial layer^68^, and the latter may stem from photo-oxidizing cytochrome *c* oxidase in the mitochondria of neurons^10,37^. Thus, changes in oscillations of both neurophysiological signals can be independent or incoherent. However, each of them plays a key role in driving changes in the electrophysiological activity of neurons (i.e., EEG power). We term this process the reversal of directional connectivity or coupling between electrophysiological and hemodynamic/metabolic activity caused by tPBM in the resting brain. In addition, either undirectional (HbO → EEG) or bidirectional enhancement (HbO ↔ EEG) by tPBM in the neurogenic band revealed that R800 tPBM enabled significant stimulation and/or reversal of the electrophysiological coupling between neuronal and hemodynamic activity (i.e., EEG ↔ HbO). This phenomenon is consistent with the mechanism of vasomotion in the neurogenic band^67^.

Second, the significant changes induced by L800 tPBM in the sham-subtracted directional connectivity in the neurophysiological network were similar to those induced by R800, as described above, with only minor differences. Specifically, in the neurogenic band, L800 augmented the directed connectivity of EEG alpha and beta powers in the ipsilateral PFC, in contrast to the lateral effect of R800.

Taken together, the overall trend of a significant reduction in MVC (CCO ↔ HbO) across both endogenic and neurogenic bands on both lateral sides of the PFC reinforces that either R800 or L800 tPBM-induced modulation in CCO and HbO oscillations is incoherent, causing desynchronization between them. The net coupling of CCO → HbO was positive in the resting brain (blue rectangles in Fig. 5), but tPBM suppressed the bidirectional couplings between them. tPBM-induced concurrent upregulation of CCO and HbO has been reported previously^10,27^, and non-directional decoupling between them in response to 1064-nm tPBM was also recently published^39^. However, the directional decoupling of MVC in both the endogenic and neurogenic bands (in particular, the directional HbO → CCO) by 800-nm tPBM is reported here for the first time. Furthermore, this study clearly demonstrated that both R800 and L800 enabled significant increases in CCO → EEG and a reversal of EEG → HbO on both the contralateral and ipsilateral sides of the PFC in the endogenic and/or neurogenic bands. The underlying mechanism or hypothesis of how tPBM modulates CCO and HbO for reversible coupling with EEG frequency-specific powers needs to be further investigated in future studies.

#### Novel construction of frequency-specific neurophysiological networks and potential applications

In this study, we investigated and constructed cerebral neurophysiological networks in three ISO frequency bands based on their physiological representations. The slowest endogenic band is associated with relaxation-contraction oscillation in the endothelium mediated by released potent vasoactive factors, such as nitric oxide (NO), prostacyclin, free radicals, endothelin, and endothelium-derived hyperpolarizing factor^12,66^. The neurogenic band corresponds to dilation-contraction cycles of vessel walls affected by neurotransmitters and vasoactive ions from neurons^67^. Finally, the relatively fast myogenic oscillation is believed to result primarily from smooth muscle cells on the vascular wall for global vasomotion^9^.

Irregularities in vasomotion have been reported and associated with aging and various neurological disorders and diseases, including Alzheimer’s disease (AD)^69^, cardiovascular disease^70^, and atherosclerosis^71^. Adverse effects of aging and AD on neurovascular coupling of the brain have also been reported^14,72^. For young healthy humans, on the other hand, cerebral vasomotion, bilateral connectivity, and unilateral MVC or physiological coupling over the prefrontal cortex at rest are evidenced relatively stable and reproducible^35,39^.

By utilizing a dual-mode brain-sensing system and multivariate coherence analysis, in this study, we were able to identify and quantify directed (effective) connectivity among three cerebral neurophysiological entities of the human PFC as potential biological or neurophysiological features of the resting human brain. These features, and in general network neurophysiology, can be further investigated and applied to different groups of clinical populations. For instance, the effects of age, neurological disorders, or other brain diseases on the proposed neurophysiological networks can be assessed for different severities or grades using the framework developed in this study. Our novel methodology also opens the door to identifying potential biomarkers for the early diagnosis of neurophysiological diseases or to monitoring the efficiency of respective treatment or rehabilitation procedures for the diseases. In addition, recently published studies have demonstrated the feasibility of using bbNIRS to identify metabolic and hemodynamic disturbances before, during, and after epileptic seizures^73^. Overall, we believe that the approach introduced in this paper is novel, significant, and useful for both future clinical applications and a better understanding of the neurophysiological networks among hemodynamic, metabolic, and electrophysiological activities in the human PFC at rest and under noninvasive tPBM.

#### Other key considerations of the study

Our long-term goal is to develop a compact, easy-to-use, and neurophysiology-measurable device with a novel analysis that can facilitate digital biomarkers for future clinical applications. One modality for quantifying hemodynamics and metabolism is bbNIRS. Taking such measurements on the forehead without any interference from hair makes the measurements significantly more portable and efficient, with a much better signal-to-noise ratio than those over the entire head, reducing both the physical and mental burden on patients. Neural physiologically, the frontal cortex plays a key role in human cognition; therefore, the directed neurophysiological networks of the frontal cortex of patients with brain disorders are likely to be abnormal. In addition, numerous studies have demonstrated significant positive effects of tPBM delivered to the human forehead^17,18, 48, 74^. Such a delivery site minimizes the uncertainty of treatment dosage without considering any heterogeneity in hair color, thickness, and density. Thus, as a “proof-of-concept”, the frontal/prefrontal cortex was chosen as a unique spatial/cortical window for us to non-invasively “view” and modulate the directed neurophysiological connectivity of the human PFC at rest and after tPBM.

Several red or NIR wavelengths are popularly used through lasers or light-emitting diodes (LEDs) in PBM and tPBM; they are 660, 800–850, 1064–1070 nm, as listed comprehensively in^21^. The rationale behind these wavelength selections is based on the light absorption of oxidized CCO and the light scattering properties of tissue in the red and NIR ranges. In theory, three factors affect the outcome of PBM/tPBM: (1) oxidized CCO has two gentle or dull light absorption peaks at 660 nm and 800–850 nm, (2) living tissue has smaller light scattering coefficients in 1064–1070 nm than 660 and 800–850 nm, meaning that 1064–1070 nm light can reach a deeper tissue region, and (3) hemoglobin within red blood cells absorbs more light at 660 nm than all other given wavelengths, implying that 660 nm light is less effective for studying or treating the human brain because of its limited penetration^75^. Taken together and given the commercial availability of laser or LED devices, 800–850 nm or 1064–1070 nm becomes the practical wavelength range available for tPBM research and studies. However, it is not clear whether both wavelength ranges result in similar physiological effects or whether one is better than the other^76^. A sizable number of studies have reported the physiological effects of a 1064-nm laser^10,27, 29, 31, 33, 36, 37, 39, 52, 77–79^. In this study, we explored the physiological effects of 800-nm tPBM, which would guide the optimal selection of wavelengths for more effective outcomes of tPBM.

### Limitations and future works

First, during the experimental setup, we did not have a standard procedure to accurately place both EEG electrodes and the bbNIRS probe holder on the participant’s head to ensure the same head location among all subjects and in different days. Second, the relatively low sampling rate (0.667 Hz) of the bbNIRS and short data collection duration (7 min pre- and post-tPBM) prevented us to achieve a high-frequency resolution, which may lead to low accuracy in spectral amplitude and coherence in the low-frequency range, especially in the endogenic band. Third, there is no gold standard connectivity analysis method for the constructed physiological network, and thus there might be a discrepancy between the coupling values obtained by the GPDC and other methods. Last, our quantified results or metrics may be potentially contaminated by extracranial layers of the human head. To minimize this potential confounding factor, extra optical channels of fNIRS with a short source-detector (S-D) separation (commonly ~ 0.8–1.2 cm) have been used for systemic noise removal in task-evoked hemodynamic studies^80–84^, where stimulating tasks activate only the cortical region. However, most fNIRS-based studies for the quantification of resting-state functional connectivity (RSFC) did not develop an appropriate methodology to remove this confounding effect until a recent report, which confirmed that RSFC can be quantified more accurately with a short S-D reading correction than without correction^85^.

As for future work, we need to develop a set of measurable metrics with respect to several facial landmarks (e.g., nasion and eyebrows) that can be used as references for accurate placement of the EEG and bbNIRS sensors. Next, to enable a longer-period and less-artifact recording from the human brain, modifications and improvements are needed on the bbNIRS setup, measurement protocol, and quantification algorithms for reducing movement artifacts and systemic/physiological noises. Also, it is necessary to consider the implementation of short-distance channels in bbNIRS for removing possible contamination of extracranial layers to the determined/interpreted results. The networks developed in this exploratory study are considered the first step in the investigation of network physiology. These neurophysiological networks can be extended to delta and theta EEG frequency bands, and the quantified features of physiological coupling can be assessed further to propose potential biomarkers for more accurate diagnose of different neurological disorders.

## Conclusion

The 7-min resting-state measurement demonstrated the unique ability of a low-cost, dual-modality, brain-sensing system analyzed using a frequency-specific multivariate model to identify and quantify neurophysiological networks among the electrophysiological, hemodynamic, and metabolic activities of the human prefrontal cortex. This novel methodology facilitates a better understanding of the mechanisms underlying neurovascular, neurometabolic, and metabolicvascular couplings and their respective directionalities in the resting human PFC. The identified effective couplings clearly illustrate the leading or driving role of oxygen demand caused by metabolic and electrophysiological activity on the supply, which is reflected by more hemodynamic activity, especially in the endogenic and myogenic bands. In addition, dual-modality measurements after 800-nm tPBM enabled the identification of frequency-specific tPBM-induced alterations of directed connectivities in neurophysiological networks. Specifically, both R800 and L800 tPBM enabled significant increases in directed coupling from metabolic to electrophysiological activity (CCO → EEG) and a reversal of the directed connectivity of the NVC (i.e., HbO → EEG) with respect to that in the resting state on both the contralateral and ipsilateral sides of the PFC in the endogenic and/or neurogenic bands. These results illustrate that tPBM delivered on either lateral side of the PFC can perturb the oxygen demand–supply balance by photo-stimulating metabolic and hemodynamic activities first, causing an increase in electrophysiological activity. These observations shed light on how tPBM affects the cerebral metabolism and hemodynamics concurrently and independently, leading to an increase in electrophysiological activity. Given the ability to comprehensively quantify neurophysiological networks in the human PFC, the novel methodology reported in this study has the potential to become a portable and noninvasive means for the detection, characterization, and monitoring of brain disorders and their respective treatment effects, including those after tPBM.

### Supplementary Information

Supplementary Information.

## Data Availability

The datasets analysed during the current study are available from the corresponding author on reasonable request.
