# Supplementary material for: Directed physiological networks in the human prefrontal cortex at rest and post transcranial photobiomodulation
Source: Sci Rep. 2024 May 3;14:10242. doi: 10.1038/s41598-024-59879-7 (PMC11068774; doi:10.1038/s41598-024-59879-7)
Supplement: Supplementary file 1 — Supplementary Information. [file 41598_2024_59879_MOESM1_ESM.docx]

Directed physiological networks in the human prefrontal cortex at rest and post transcranial photobiomodulation

Sadra Shahdadian, Xinlong Wang, Hanli Liu

**Supplementary Materials**

**A. Theoretical Foundation for conversion from ΔOD(t, λ) to Δ[HbO](t, λ) and Δ[CCO](t, λ)**

Methods to quantify changes in concentrations of oxygenated hemoglobin (Δ[HbO]), deoxygenated hemoglobin (Δ[HHb]), and redox-state cytochrome c oxidase (Δ[CCO]) have been developed and reported (Kolyva, Tachtsidis et al. 2012, Wang, Tian et al. 2017). A brief review is provided below for general readers who wish to understand the theoretical foundation and processing methods in depth. Figure S1 illustrates graphically the processing steps, which are described below.

**Steps 1 and 2:**

A broadband near-infrared spectroscopy (bbNIRS) system provides measurements of optical spectra at different times (t), as expressed *I(t, λ)*. A relative optical density spectrum, *ΔOD*(*t, λ*), can be defined and calculated at each wavelength λ as (Kolyva, Tachtsidis et al. 2012, Wang, Tian et al. 2017):

$\Delta OD(t,\lambda)={log}_{10} [\frac{I_{0}(t=0, \lambda)}{I(t,\lambda)}]$, (1)

where *I_0_(t=0, λ)* can be the baseline spectrum at time *t*=0 or an average of several initial baseline spectral readings (i.e., the first two spectra collected in each experiment), and *I(t, λ)* represent time-varying spectra acquired at each time point throughout the entire experiment.

**Step 3**:

The estimations of Δ[HbO] and Δ[CCO] from raw spectral data taken with bbNIRS throughout the experiment were based on modified Beer-Lambert’s law (Kocsis, Herman et al. 2006), which offers a quantitative relationship of *ΔOD(λ)* on *Δ[HbO], Δ[HHb], and Δ[CCO]* at each wavelength, *λ*, at each time point, with a wavelength-dependent path-length factor, *L(λ)*. Based on optical diffusion theory (Wang, Tian et al. 2016), *ΔOD(λ)*/*L(λ)* can be expressed as a sum of optical absorbance contributed by *Δ[HbO]*, *Δ[HHb],* and *Δ[CCO]* components, as given in eq. (2).


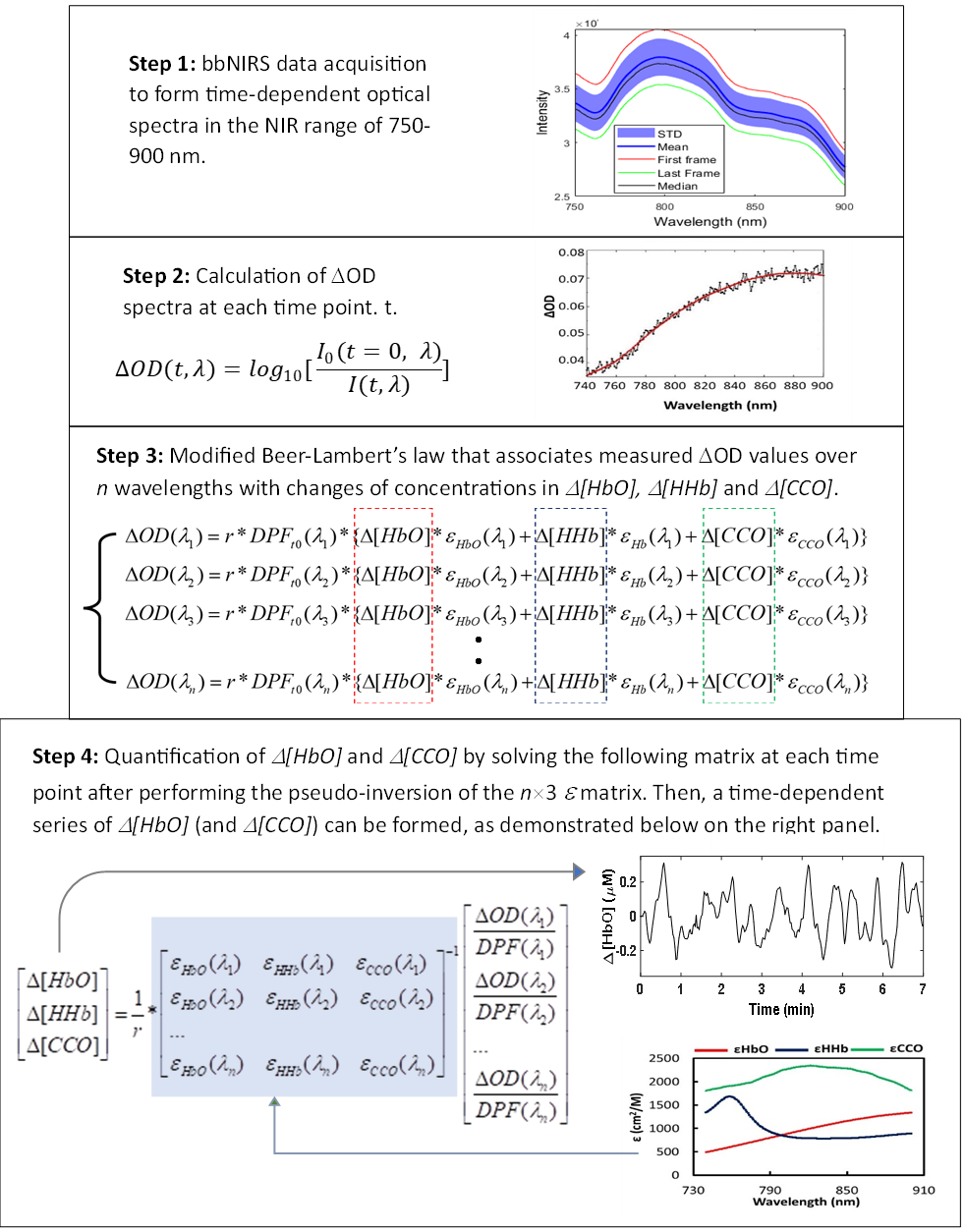


**Figure S1**. A data processing flow chart used to quantify *Δ[HbO]* and *Δ[HHb]* from raw bbNIRS data.

$\left[\begin{matrix} \frac{\Delta OD(\lambda_{1})}{L(\lambda_{1})} \\ \frac{\Delta OD(\lambda_{2})}{L(\lambda_{2})} \\ \frac{\Delta OD(\lambda_{3})}{L(\lambda_{3})} \\ ... \\ \frac{\Delta OD(\lambda_{n})}{L(\lambda_{n})} \end{matrix}\right]=\Delta[HbO]*\left[\begin{matrix} \varepsilon_{HbO}(\lambda_{1}) \\ \varepsilon_{HbO}(\lambda_{2}) \\ \varepsilon_{HbO}(\lambda_{3}) \\ ... \\ \varepsilon_{HbO}(\lambda_{n}) \end{matrix}\right]+\Delta[HHb]*\left[\begin{matrix} \varepsilon_{HHb}(\lambda_{1}) \\ \varepsilon_{HHb}(\lambda_{2}) \\ \varepsilon_{HHb}(\lambda_{3}) \\ ... \\ \varepsilon_{HHb}(\lambda_{n}) \end{matrix}\right]+\Delta[CCO]*\left[\begin{matrix} \varepsilon_{CCO}(\lambda_{1}) \\ \varepsilon_{CCO}(\lambda_{2}) \\ \varepsilon_{CCO}(\lambda_{3}) \\ ... \\ \varepsilon_{CCO}(\lambda_{n}) \end{matrix}\right]$, (2)

where *Δ[HbO], Δ[HHb] and Δ[CCO]* are relative concentration changes of HbO, HHb and CCO respectively; *ε_HbO_(λ)*, *ε_HHb_(λ)* and *ε_CCO_(λ)* represent the extinction coefficients at each wavelength of HbO, HHb and CCO, which can be found in ref. (Kolyva, Tachtsidis et al. 2012); *L(λ)* is a wavelength dependent factor that denotes the effective pathlength of the detected photons through tissues at each wavelength. Furthermore, according to the Modified Beer-Lambert Law (Kocsis, Herman et al. 2006, Scholkmann, Kleiser et al. 2014), *L(λ)* can be expressed as:

,

(3)

where *r* is a constant that denotes the source-detector distance. In this study, we used source detector separation of 3 cm, so *r*=3. The wavelength dependence of *L(λ)* is caused by a wavelength-dependent differential pathlength factor, *DPF(λ)*.

**Step 4**:

By substituting Eq. (3) into Eq. (2) for multiple wavelengths, the estimation of *Δ[HbO], Δ[HHb] and Δ[CCO]* can be expressed as follows:

$\left[\begin{matrix} \Delta[HbO] \\ \Delta[HHb] \\ \Delta[CCO] \end{matrix}\right]=\frac{1}{r}*\left[\begin{matrix} \varepsilon_{HbO}(\lambda_{1}) \varepsilon_{HHb}(\lambda_{1}) \varepsilon_{CCO}(\lambda_{1}) \\ \varepsilon_{HbO}(\lambda_{2}) \varepsilon_{HHb}(\lambda_{2}) \varepsilon_{CCO}(\lambda_{2}) \\ ... \\ \varepsilon_{HbO}(\lambda_{n}) \varepsilon_{HHb}(\lambda_{n}) \varepsilon_{CCO}(\lambda_{n}) \end{matrix}\right]^{-1}\left[\begin{matrix} \frac{\Delta OD(\lambda_{1})}{DPF(\lambda_{1})} \\ \frac{\Delta OD(\lambda_{2})}{DPF(\lambda_{2})} \\ ... \\ \frac{\Delta OD(\lambda_{n})}{DPF(\lambda_{n})} \end{matrix}\right]$. (4)

In order to accurately solve *Δ[HbO], Δ[HHb] and Δ[CCO]* using Eq. (4), we would need to know *DPF(λ)* in the wavelength range of our measurements. It is known that appropriate or accurate selection/estimation of wavelength-dependent DPF is crucial for accurate estimation of chromophore concentrations (Matcher, Cope et al. 1994). In this study, DPF(λ) values were assumed to be time-invariant because of given stable brain optical properties. Based on diffusion theory with the semi-infinite boundary geometry (Fantini, Hueber et al. 1999), *DPF(λ)* can be determined by

(5)

where *µ_a_(λ)* and *µ_s_'(λ)* are the estimated absorption and reduced scattering coefficients across the wavelength range of interest.

Values of *µ_a_(λ)* and *µ_s_'(λ)* were measured using a tissue oximeter (OxiplexTS, ISS) that operates in the frequency-domain. This device provides readings of *µ_a_* and *µ_s_'* values at 750 nm and 830 nm, as well as absolute concentrations of [HbO] and [HHb] (Fantini, Hueber et al. 1999). However, to obtain *µ_s_'(λ)* values across the entire range of wavelengths from 780-900 nm, we used Mie theory to interpolate and extrapolate the two measured *µ_s_'* values at 750 nm and 830 nm. Mie theory is typically represented by *kλ^-b^*, where *k* and *b* were determined by fitting this equation to both *µ_s_'* values at 750 nm and 830 nm (Jacques 2013). In addition, absorption coefficients in the same wavelength range (780-900 nm) were estimated based on [HbO] and [HHb] measured by the same tissue oximeter (Wang, Tian et al. 2016).

After combining the measured *ΔOD(λ)* values across the measurement period and empirical *µa(λ)* and *µs'(λ)* values of the human forehead (Wang, Tian et al. 2017), we were able to solve eq. (4) at each measurement time point using MATLAB, achieving temporal series of *Δ[HbO], Δ[HHb]* and *Δ[CCO]* under respective experimental conditions, as shown in Step 2 of Fig. 6 in the main paper. Specifically, our calculations covered the spectral range of 780-900 nm with a total of 121 wavelengths.

**B. Decomposition of a [HbO] Time Series into Three Frequency Bands**

Figure S2(a) below shows an example of the Δ[HbO] time series from one of the two-channel bbNIRS datasets of a subject. Three curves in Figure S2(b) were obtained after applying a Butterworth band-pass filter to the trace in Figure S1(a) using separate bandpass filters in endogenic (E: 0.005-0.02 Hz), neurogenic (N: 0.02-0.04 Hz), and myogenic (M: 0.04-0.2 Hz) frequency ranges. This set of figures illustrates how different infraslow oscillation (ISO) components contribute to the composition of the wideband (0.005–0.2 Hz) original signal, as shown in Figure S1(a).


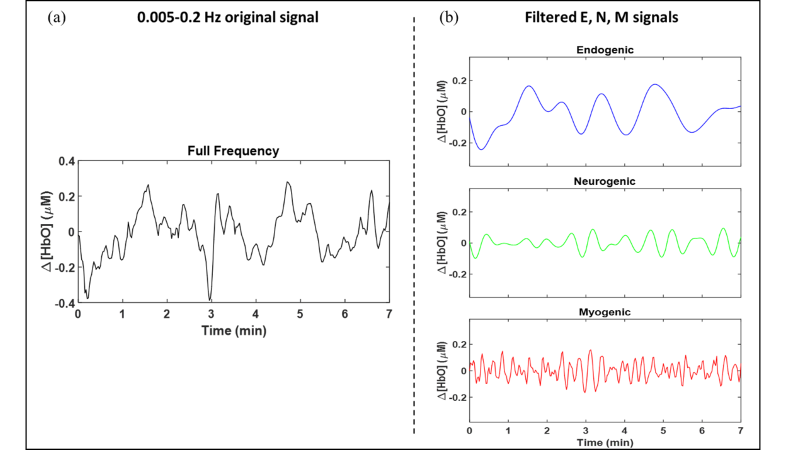


**Figure S2** (a) A 7-min time series of Δ[HbO] derived from one of the 2-channel bbNIRS data sets from a random participant. The three panels in (b) on the right were obtained after Butterworth band-pass filtering of the original signal in the three predefined E/N/M bands, namely, 0.005-0.02 Hz, 0.02-0.04 Hz, and 0.04-0.2 Hz, respectively.

**C. From MVAR model to PDC and GPDC**

As described in step (3) of ‘Overview of data processing steps’, the time-domain representation of the MVAR model takes the form of

$\boldsymbol{X}\left( t \right)=\sum_{n=1}^{p} \boldsymbol{A}\left( n \right)\boldsymbol{X}(t-n)\boldsymbol{E}\left( t \right),$ (S1)

where ***A*** is the *k* × *k*-sized matrix of coefficients with *A_ij_*(*n*) as elements, ***E***(*t*) is a prediction error vector of size *k*, and *p* is the order of the model (Blinowska 2011). The frequency-domain representation of the MVAR can be obtained by changing the sign of *A* and applying the Z-transform to (S1):

***E****(f) =* ***A****(f)* ***X****(f),* (S2)

where:

$\boldsymbol{A}(f)=\sum_{m=0}^{p} \boldsymbol{A}(m)e^{-2\pi imf\Delta t}$. (S3)

Accordingly, the PDC from source *j* to sink *i* can be defined as (Baccalá and Sameshima 2001):

$PDC_{j,i}(f)=\frac{A_{ij}(f)}{\sqrt{\sum_{n=1}^{k} A_{nj}(f)A_{nj}^{*}(f)}}$, (S4)

where the term of $\sum_{n=1}^{k} A_{nj}(f)A_{nj}^{*}(f)$ represents the MVAR model coefficients in the *j*th column of ***A***(*f*) multiplied by their complex conjugate transpose. As shown in (S4), the PDC is normalized to show a ratio between the outflow from channel *j* to channel *i* to all the outflows from the source channel *j*. Thus, it emphasizes the sinks rather than the sources (Blinowska 2011).

To change the normalization factor from sink to source, Baccala et al. (Baccala, Sameshima et al. 2007) proposed the GPDC from source *j* to sink *i* as follows:

$GPDC_{j,i}(f)=\frac{\frac{1}{\sigma_{i}}A_{ij}(f)}{\sqrt{\sum_{n=1}^{k} \frac{1}{\sigma_{n}^{2}}A_{nj}(f)A_{nj}^{*}(f)}},$ (S5)

where $\sigma_{n}^{2}$ refers to the variance of prediction error of the *n*th time series (i.e., *E_n_*(*t*)).

As examined by Schelter et al.(Schelter, Timmer et al. 2009), PDC has some disadvantages compared to GPDC; namely: (1) PDC is sensitive to the number of signals emitted from a given source, (2) PDC is dependent on the units of measurement of the source and target processes, and (3) PDC shows the ratio between the outflow from channel *j* to channel *i* to all the outflows from the source channel *j*; thus, it does not represent the absolute strength of the connectivity.

**References**

Baccalá, L. A. and K. Sameshima (2001). "Partial directed coherence: a new concept in neural structure determination." Biological cybernetics **84**(6): 463-474.

Baccala, L. A., K. Sameshima and D. Y. Takahashi (2007). Generalized partial directed coherence. 2007 15th International conference on digital signal processing, Ieee.

Blinowska, K. J. (2011). "Review of the methods of determination of directed connectivity from multichannel data." Med Biol Eng Comput **49**(5): 521-529.

Fantini, S., D. Hueber, M. A. Franceschini, E. Gratton, W. Rosenfeld, P. G. Stubblefield, D. Maulik and M. R. Stankovic (1999). "Non-invasive optical monitoring of the newborn piglet brain using continuous-wave and frequency-domain spectroscopy." Phys Med Biol **44**(6): 1543-1563.

Jacques, S. L. (2013). "Optical properties of biological tissues: a review." Phys Med Biol **58**(11): R37-61.

Kocsis, L., P. Herman and A. Eke (2006). "The modified Beer-Lambert law revisited." Phys Med Biol **51**(5): N91-98.

Kolyva, C., I. Tachtsidis, A. Ghosh, T. Moroz, C. E. Cooper, M. Smith and C. E. Elwell (2012). "Systematic investigation of changes in oxidized cerebral cytochrome c oxidase concentration during frontal lobe activation in healthy adults." Biomed Opt Express **3**(10): 2550-2566.

Matcher, S. J., M. Cope and D. T. Delpy (1994). "Use of the water absorption spectrum to quantify tissue chromophore concentration changes in near-infrared spectroscopy." Phys Med Biol **39**(1): 177-196.

Schelter, B., J. Timmer and M. Eichler (2009). "Assessing the strength of directed influences among neural signals using renormalized partial directed coherence." Journal of neuroscience methods **179**(1): 121-130.

Scholkmann, F., S. Kleiser, A. J. Metz, R. Zimmermann, J. Mata Pavia, U. Wolf and M. Wolf (2014). "A review on continuous wave functional near-infrared spectroscopy and imaging instrumentation and methodology." Neuroimage **85 Pt 1**: 6-27.

Wang, X., F. Tian, D. D. Reddy, S. S. Nalawade, D. W. Barrett, F. Gonzalez-Lima and H. Liu (2017). "Up-regulation of cerebral cytochrome-c-oxidase and hemodynamics by transcranial infrared laser stimulation: A broadband near-infrared spectroscopy study." J Cereb Blood Flow Metab **37**(12): 3789-3802.

Wang, X., F. Tian, S. S. Soni, F. Gonzalez-Lima and H. Liu (2016). "Interplay between up-regulation of cytochrome-c-oxidase and hemoglobin oxygenation induced by near-infrared laser." Sci Rep **6**: 30540.
